# Intra-operative extracorporeal irradiation of tumour-invaded craniotomy bone flap in meningioma: a case series

**DOI:** 10.1007/s00701-024-06126-7

**Published:** 2024-05-24

**Authors:** William H. Cook, Katherine Burton, Sarah J. Jefferies, Simon L. Duke, Rajesh Jena, Neil G. Burnet, Ramez W. Kirollos, Adel E. Helmy, Thomas Santarius

**Affiliations:** 1 Division of Neurosurgery, Department of Clinical Neurosciences, University of Cambridge, Cambridge, UK; 2 Department of Radiation Oncology, Cambridge University Hospitals NHS Foundation Trust, Cambridge, UK; 3 Department of Oncology, Cambridge University Hospitals NHS Foundation Trust, Cambridge, UK; 4 Department of Oncology, University of Cambridge, Cambridge, UK; 5 Present Address: The National Neuroscience Institute, Tan Tock Seng, Singapore; 6 Present Address: Proton Beam Therapy Centre, The Christie NHS Foundation Trust, Manchester, UK

**Keywords:** Case series, Meningioma, Radiation oncology, Skull neoplasms

## Abstract

**Background:**

Extracorporeal irradiation of tumorous calvaria (EITC) can be performed to restore function and form of the skull after resection of bone-invasive meningioma. We sought to examine the rate of tumour recurrence and other selected outcomes in patients undergoing meningioma resection and EITC.

**Methods:**

Retrospective single-centre study of adult patients undergoing meningioma resection and EITC between January 2015 and November 2022 at a tertiary neurosurgical centre. Patient demographics, surgery data, tumour data, use of adjuvant therapy, surgical complications, and tumour recurrences were collected.

**Results:**

Eighteen patients with 11 (61%) CNS WHO grade 1, 6 (33%) grade 2, and 1 (6%) grade 3 meningiomas were included. Median follow-up was 42 months (range 3–88). Five (28%) patients had a recurrence, but none were associated with the bone flap. Two (11%) wound infections requiring explant surgery occurred. Six (33%) patients required a further operation. Two operations were for recurrences, one was for infection, one was a washout and wound exploration but no evidence of infection was found, one patient requested the removal of a small titanium implant, and one patient required a ventriculoperitoneal shunt for a persistent CSF collection. There were no cases of bone flap resorption and cosmetic outcome was not routinely recorded.

**Conclusion:**

EITC is feasible and fast to perform with good outcomes and cost-effectiveness compared to other reconstructive methods. We observed similar recurrence rates and lower infection rates requiring explant compared to the largest series of cranioplasty in meningioma. Cosmetic outcome is universally under-reported and should be reported in future studies.

## Introduction

Surgery is the main mode of treatment of most meningiomas and the degree of resection is predictive of recurrence [26]. While there have been efforts to refine Simpson grading [10], its grades are still used widely when resecting meningiomas, including bone-invasive meningiomas. Excision of affected bone is part of the grading system, reflecting the increased risk of recurrence associated with meningioma remaining in bone. Therefore, when treating bone-invasive meningiomas it is important to achieve not only maximum safe tumour resection but also functionally sound and cosmetically acceptable reconstruction of the skull. An increasing range of materials, including polymethyl methacrylate (PMMA) [31], titanium [5], polyetheretherketone (PEEK) [3], allogeneic bone [29], and other methods have been developed to carry out such reconstructions. These include pre-manufactured individualized implants [5], and generic materials, including PMMA and titanium mesh, that can be adapted by the surgeon intraoperatively. In cases where the bone is infiltrated but its architecture and mechanical strength largely preserved, reimplantation of the bone flap after its treatment in the autoclave [30], or with high dose radiotherapy, termed extracorporeal irradiation of tumorous calvaria (EITC), could be an alternative [28]. However, little is known about EITC’s functional efficacy and safety as to date only 23 cases have been reported in total [13, 16, 18, 28]. Furthermore, despite the utility of these techniques, each have their own complication profiles due to the technique itself or the material used.

EITC may be a useful adjunct to cranioplasty techniques and may provide a more cost-effective method of skull reconstruction with good cosmetic outcome and minimal chance of immunological rejection when compared to foreign implants [30]. The aim of the present study is to add to the collective experience by reporting the use of EITC in 18 consecutive patients with bone-invasive meningioma including the tumour characteristics of each case and their long-term results.

## Methods

### Patient selection

A retrospective single-centre review was conducted of consecutive adult patients undergoing meningioma resection and EITC between January 2015 and November 2022 at a tertiary neurosurgical centre. This case series has been reported in line with the PROCESS Guideline [1]. The study protocol was review by the hospital research and development office and, as this was a retrospective review of case notes, no study-specific consent, i.e. ‘research consent', was mandated. In each case, a patient-specific consent was obtained for treatment (‘clinical consent’), as per established routine clinical practice. One patient gave consent to the publication of their clinical photographs.

### EITC technique

The decision to reconstruct the skull defect with the autologous bone and EITC was anticipated preoperatively based on imaging (CT and/or MRI with contrast) but confirmed intraoperatively once the bone flap was raised and tumour invasion was macroscopically inspected. A rongueur or high-speed drill were used to remove exostotic tumour-infiltrated bone and/or frank tumour. Bone flaps were then wrapped in wet gauze, placed in a sterile plastic bag and a container, and transferred to the Radiotherapy Department. The bone flap was placed on the linear accelerator treatment couch and tissue equivalent material (bolus) used to form a square block around the sterile container. The treatment was delivered using a single, direct applied with 6-MV photon beam to a minimum dose of 80 Gy. Radiation delivery time was approximately 30 min with the total process taking approximately 45 min. After the intracranial tumour resection was completed the irradiated bone flap was reimplanted and fixed with standard titanium metalware; in 8/18 cases small pieces of titanium mesh were used to cover a bone defect. The surgical wound was closed in a standard fashion.

### Data collection

Demographic and clinical information was collected from hospital electronic medical records. The collected variables were patient age, sex, tumour characteristics (CNS WHO grade, histological subtype, Ki-67 index, laterality, location), surgery characteristics (Simpson grade), follow-up characteristics (time to last follow-up, rates of tumour recurrence, cosmetic outcome, adjuvant therapy, death), and complications (infection, bone resorption, reoperation).

### Statistical analysis

Data were compiled in Microsoft® Excel (version 16.73; Microsoft®, Redmond, WA, USA) and summary statistics and figures produced where possible.

## Results

Eighteen consecutive patients with bone-invasive meningioma have undergone EITC at our institution between January 2015 and November 2022 (Table 1). The mean age of these patients was 59 ± 16 years (range 18 to 78), with 12 females (67%) and 6 males (33%) included.

**Table 1:** Clinical and demographic data of patients undergoing EITC in chronological order of surgery

Age at Surgery (yrs), Sex		CNS WHO Grade	Location	Radial dimension of bone changes (%)	Transverse dimension of bone changes	Frank tumour identified	Involvement of extracranial soft tissue	Simpson grade	Supplementary allograft	Adjuvant therapy	Follow-up (mos)	OS (mos)	Comments
74, M	Recurrent	3	Parasagittal	< 25	Beyond outer table	Yes	No	2	Titanium mesh	None, previous radiotherapy	14	31	Died 31 mos post-op, also required explant surgery for infection after second operation
76, M	Primary	1	Parasagittal	< 25	Inner table	No	No	2	None	None	26	62	Died 62 mos post-op, rapidly progressing Parkinson’s disease
48, M	Primary	1	Falcotentorial	25–50	Inner table	Yes	No	2	Titanium mesh	EBRT	88	88	
34, F	Primary	2	Convexity	< 25	Diploe	Yes	No	1	None	None	78	78	
45, F	Primary	2	Parasagittal	25–50	Beyond outer table	Yes	No	4	Titanium mesh	EBRT	47	47	
63, F	Primary	2	Spheno-orbital/convexity	50–75	Beyond outer table	Yes	Yes	4	None	EBRT	47	47	
48, F	Primary	1	Parasagittal	25–50	Beyond outer table	Yes	No	3	Titanium mesh	EBRT	56	56	
62, M	Primary	1	Parasagittal	50–75	Beyond outer table	Yes	No	2	None	None	50	50	Post-op infection requiring explant and delayed cranioplasty
66, F	Primary	2	Convexity	25–50	Beyond outer table	Yes	Yes	4	None	EBRT, chemotherapy (Bevacizumab)	44	45	Died 45 mos post-op
71, M	Primary	2	Parasagittal	25–50	Beyond outer table	Yes	Yes	4	Titanium mesh	EBRT	47	47	Advanced colorectal cancer, wound breakdown after radiotherapy—titanium mesh was removed to facilitate healing
61, F	Primary	1	Convexity	50–75	Beyond outer table	Yes	No	1	None	None	42	42	
57, F	Primary	1	Convexity	25–50	Beyond outer table	Yes	No	1	None	None	41	41	
78, F	Primary	1	Parasagittal	< 25	Beyond outer table	Yes	No	4	Titanium mesh	None	21	21	
47, F	Primary	1	Spheno-orbital/convexity	25–50	Outer table	No	No	2	Titanium mesh	None	20	20	
18, M	Primary	1	Parasagittal	< 25	Diploe	Yes	No	2	None	None	14	14	
75, F	Recurrent	2	Parasagittal	< 25	Beyond outer table	Yes	Yes	4	Titanium mesh	EBRT	15	15	
54, F	Primary	1	Parasagittal	50–75	Beyond outer table	Yes	No	4	None	None	12	12	Radiation-induced multiple meningiomas
76, F	Primary	1	Spheno-orbital/convexity	25–50	Beyond outer table	Yes	No	3	None	None	3	3	

*mos*, months; *EITC*, extracorporeal irradiation of tumorous calvaria; *OS*, overall survival; *EBRT*, external beam radiation therapy

### Tumour characteristics

All tumours in this series were histologically-confirmed meningiomas. Three patients had previously had meningiomas resected. Two patients had previous resections of tumours in different locations. There were 11 (61%) CNS WHO grade 1, 6 (33%) grade 2, and 1 (6%) grade 3 meningioma(s). Mean Ki-67 index was 8.2 ± 7.0%. All patients had preoperative CT scans and all but one patient had preoperative MRI scans. Tumour locations included 10 parasagittal, 4 convexity, 3 spheno-orbital/convexity, and 1 falcotentorial. In 4 cases there was intra-operatively apparent macroscopic involvement of the scalp.

### Surgical characteristics

**Fig. 1:**
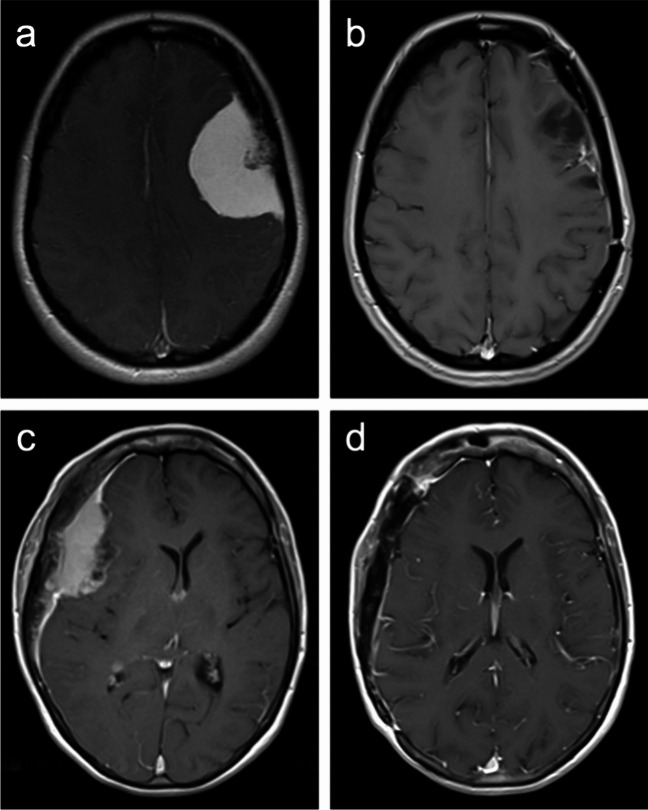
Case 4 (a&b): a, Preoperative T1-weighted MR image after contrast administration (T1 + c) in a 34-year-old woman with a CNS WHO grade 2 chordoid meningioma. There is evidence of tumour invasion into the calvaria. b, T1 + c MRI from the analogous location obtained 55 months post-operatively, demonstrating a section of the bone flap that had undergone EITC with no recurrence or bone resorption. Case 6 (c&d): c, Preoperative T1 + c MRI in a 63-year-old woman with an atypical meningioma. There is evidence of tumour invasion into the calvaria. d, T1 + c MRI from the analogous location obtained 48 months post-operatively, demonstrating a section of the bone flap that had undergone EITC with no recurrence or bone resorption

**Fig. 2:**
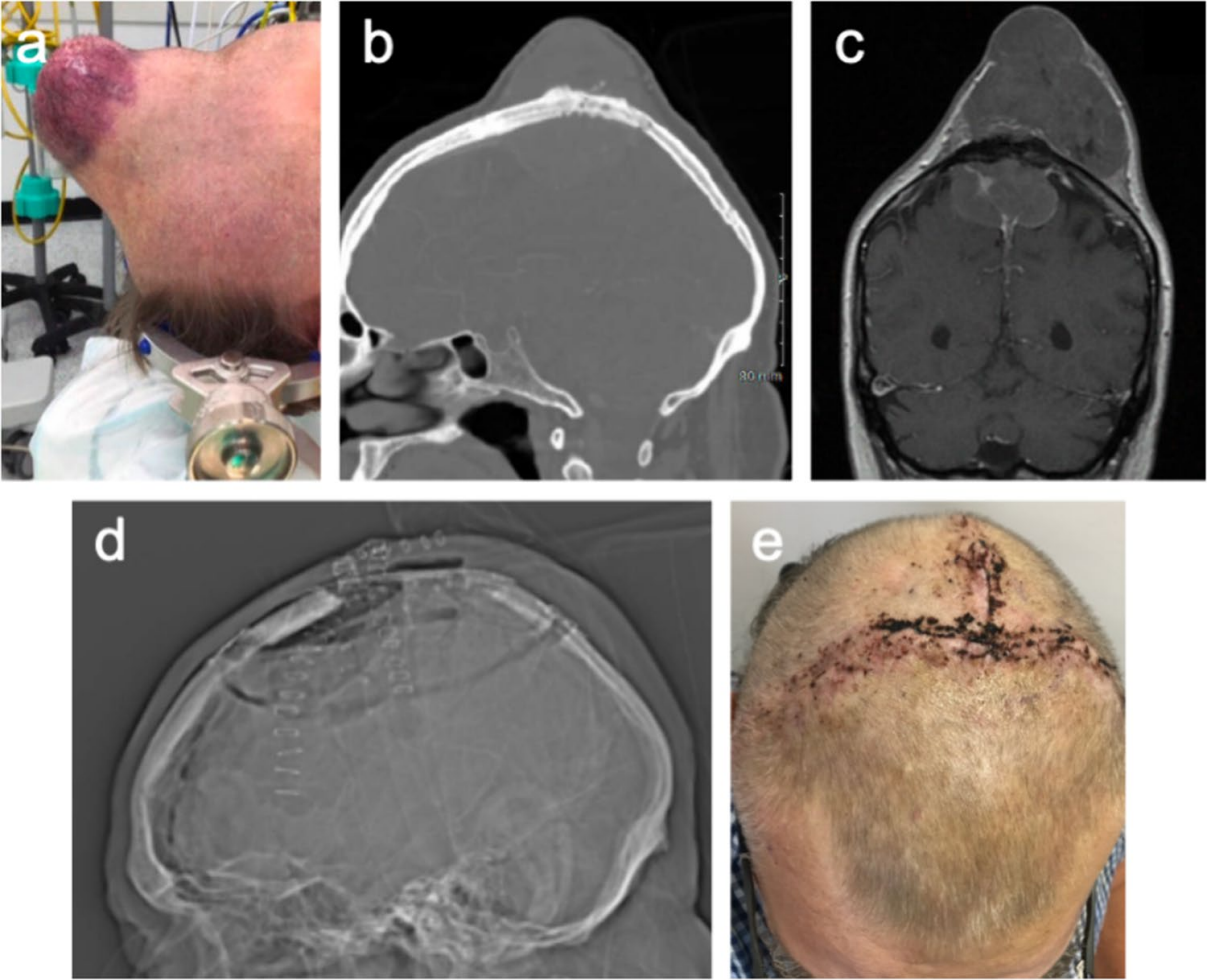
This 71-year-old man was reluctant to undergo treatment of his parasagittal meningioma, hiding under his hat a tumour that had grown through the bone and into the scalp. a, Preoperative photograph. b, Preoperative CT scan. c, Preoperative MRI scan. He underwent resection and reconstruction with EITC and primary skin closure in one sitting. The centre of the bone flap was replaced with tumour and was excised. This defect was covered with a titanium mesh. This was a CNS WHO grade 2 meningioma with Simpson grade 4 resection. The patient underwent external beam radiation therapy. Approximately 12 months after surgery the wound started to dehisce over the titanium mesh and the titanium was removed, which allowed the skin to heal. d, Post-operative CT scout image demonstrating bone and implant appearances. e, Post-operative skin appearances

Three (17%) operations were Simpson grade 1 resections, six (33%) were Simpson 2, two (11%) was Simpson 3, and seven (39%) were Simpson 4. Only one out of four convexity tumours, compared to 5 out of 10 parasagittal tumours, were Simpson grade 4 resections. In all cases, frank tumour was removed with rongueur and/or drill, and in all cases the margin of bony resection was at least 10 mm. The degree of tumour infiltration of the irradiated and retained bone flap in terms of transverse (across the thickness of the bone) and lateral (across the width of the bone) extent in each individual case is listed in Table 1. In two, two, and one case(s) the tumour reached the inner table, diploe, and outer table, respectively. The meningioma grew beyond the outer table in the remaining 13 patients. In terms of lateral extent, six cases had < 25% of the bone flap infiltrated (as per pre-operative imaging), while eight had 25–50%, and four had 50–75%. A minimum of 80 Gy of radiation was delivered to each patient’s tumorous calvaria. Figure 1 presents two meningiomas whose management included EITC and their long-term radiological appearances. Figure 2 presents another large bone- and skin-invasive meningioma that underwent EITC as part of surgery and post-operative appearances. Seven (39%) patients received adjuvant therapy, six receiving radiation therapy, one receiving stereotactic radiosurgery, and one patient who received radiation therapy also received bevacizumab.

### Tumour control and patient survival

Median follow-up was 42 months (range 3 to 88). Five (28%) patients had a recurrence, but none were associated with the bone flap. Of these patients, three had intracranial recurrences and two had bone recurrences away from the bone flap site. The other thirteen (72%) patients did not have a recurrence. Of those tumours that did recur, mean time to recurrence was 30 ± 18 months (range 10 to 55 months). Two recurrences occurred in CNS WHO grade 1 tumours, another two were grade 2, and one was grade 3. Simpson grades in these cases ranged from 2 to 4. There were three deaths, of which two were related to progressive meningioma; one CNS WHO grade 3 and Simpson 2; the other WHO grade 2, Simpson 4). Of the patients who died, mean post-operative survival was 46 ± 13 months (range 31 to 62 months).

### Complications

Three (17%) patients developed a post-operative infection. Two (11%) wound infections requiring explant surgery occurred although neither was directly related to the bone flap; in one case there was wound dehiscence, and in the other case (Case 1) the skin flap had been previously irradiated. In the third case there was a suspicion of CSF infection (high CSF polymorph count, no organism seen). This resolved with intravenous antibiotics and did not require repeat surgery. One additional case developed titanium mesh erosion through the wound following radiation treatment. The mesh was removed to facilitate healing. There were no cases of bone flap resorption. Six (33%) patients required a further operation. Two operations were for recurrences, one was for infection, one was a washout and wound exploration but no evidence of infection was found, one patient requested the removal of a small titanium implant, and one patient required a ventriculoperitoneal shunt for a CSF collection. We found no records of explicit or inferred cosmetic issues, but cosmetic outcomes of surgery were not routinely reported.

## Discussion

Calvarial reconstruction following meningioma resection is important for structural and cosmetic reasons. Achieving good reconstructive results is made more challenging if there is tumour invasion of the calvaria, in which case reconstruction may require a partial or entire replacement of the craniotomy flap with a variety of materials [5, 31], autologous or allogeneic bone grafting [29], autoclaving [30], or irradiating the autologous bone flap. To date only 23 cases of extracorporeal irradiation of tumour-infiltrated calvarial (EITC) flap have been published [13, 16, 18, 28].

Here we present our experience with extracorporeal irradiation of a craniotomy bone flap intra-operatively, during resection of meningiomas that invaded the bone flap. This is a retrospective series of 18 patients meningiomas with a median follow-up of 42 months. In 14 cases the tumour infiltration was limited to the bone flap (convexity and parasagittal), while in 4 cases it extended beyond the bone flap (lateral sphenoid wing/spheno-orbital). CNS WHO grades higher than grade 1 may be over-represented (40%) but this is in keeping with other series of bone invasive meningioma requiring calvarial reconstruction [17, 28], and likely reflects the more aggressive behaviour of higher grade meningiomas.

### Complications of EITC

Bone resorption was one of the anticipated complications and was reported to some degree in all of the four first reported cases of EITC when 30,000 Gy was delivered [16]. However, bone resorption has not occurred in the present series nor any of the more recently published cases of EITC for meningioma when up to 120 Gy was used [13, 18, 28]. This is in contrast to series of autoclaved bone which report resorption rates of up to 19% [30], and reduced torsional strength in an animal model [14]. There were no cases of infection reported in previously published series [13, 28], but a proportion of craniotomies for tumours will unfortunately develop infection requiring craniotomy flap explantation, whether this was a primary untreated bone (2% [21]), autoclaved bone (up to 4% [24]), bone autologous graft (up to 5% [9]), or an allograft (Table 2, up to 17% [17]). Table 2 summarises studies of cranioplasty for bone-invasive meningioma [3, 6, 12, 15, 17, 19, 22]. One of our cases underwent removal of the titanium mesh in the absence of infection (Case 3). This was probably due to scar contraction caused by subsequent external beam radiotherapy leading to erosion of the titanium centre through the bone. The titanium had to be removed to allow the skin to heal. Explantation of two bone flaps for infection was required in two cases in our series (11%) which is rather high, however the total number of cases was too low to draw any meaningful conclusion. However, it seems that titanium is irritating to the scalp, especially if this is preceded or followed by radiotherapy, and smoother contour of a cranioplasty may be a better option, at least in some cases. Nevertheless, this complication is not unique to EITC and is known to occur in other types of solutions to calvarial bone loss [17]. Clearly, the number of published cases of EITC is currently insufficient to draw firm conclusions regarding infection and other complications, other than stating that complications do occur with EITC.

**Table 2:** Summary of studies reporting cranioplasty outcomes in bone-invasive meningioma

Authors & Year	Study design	Material	No. of patients	Follow-up (mos)	Complication(s)
Kotwica, et al [15]	Case report	Polypropylen-Polyester (“Codubix”)	1	36	None
Kasprzak, et al [12]	Retrospective	Polypropylen-Polyester (“Codubix”)	5 (with meningioma)	> 6*	Infection requiring explant (1)†, broken implant following trauma (1)†
Marbacher, et al [19]	Retrospective	PMMA	6	3	None
Morales-Gómez, et al [22]	Retrospective	PMMA	3 (with meningioma)	6	None
Bianchi, et al [3]	Retrospective	PEEK	6	20	Speech disturbance (1)
Lönnemark, et al [17]	Retrospective	Calcium phosphate titanium OR porous polyethylene OR (PMMA)	39 (with meningioma)	7.2‡	Tumor recurrence (15), infection requiring explant (8), fluid collection requiring drain (8), hematoma requiring surgery (2)
Clynch, et al [6]	Retrospective	Prefabricated PMMA, titanium, HA, intraoperatively designed titanium, PMMA, combined titanium and PMMA	33	34	Pseudomeningocele (4), sensory disturbance (4), seizure (4), CSF leak (2), hematoma (2), wound infection (1), cosmetic complaint (1), dysphasia (1), wound erosion (1), reoperation (5)

PMMA, polymethylmethacrylate; PEEK, polyether ether ketone; HA, hydroxyapatite

*, Follow-up duration of patients with meningioma not specified but was at least six months; †, patients who had complications were not stratified by cranioplasty indication i.e. meningioma. This series included a total of 48 patients with various cranioplasty indications; ^‡^, Mean follow-up duration not described but median time to implant failure was 220 days (7.2 months). This series also included a total of 48 patients with various neoplasms and complications were reported for all patients combined

### Tumour recurrence

There have been no bone flap-related meningioma recurrences in any of the 41 EITC cases published thus far, including the present series [13, 16, 18, 28]. The other largest series of EITC in meningioma followed up patients for a mean of 41 months and in addition to 18 patients with meningioma, treated two patients with dural-based B-cell lymphoma and one with a lung cancer metastasis [28]. One patient in their series died one month postoperatively but the authors did not describe their cause of death. As in our series, one patient in their series also required a repeat operation to remove some prominent metal hardware. Although a dose of 120 Gy was used for the EITC protocol in their series, more than the minimum of 80 Gy given at our institution, this was not associated with a higher rate of complications such as bone resorption. It is notable that tumorous long bones have been completely cleared of viable osteosarcoma tumour cells with 50 Gy [27], suggesting that higher doses may not be necessary despite the anoxic irradiation conditions, while increasing the risk of radiation-related complications [23]. Another single case of EITC has reported good outcomes 29 months following surgery for an anterior skull base meningioma with frontal bone extension [13]. In addition to meningioma, EITC has been successful in a case of calvarial sarcoma [25], supporting its use in a range of bone-invasive neoplasms affecting the calvarium.

### Alternative reconstructive methods

Meningiomas with substantial infiltration of calvaria may be better reconstructed with alternative methods. EITC is probably not ideal where there is a large amount of bone loss due to bone replacement by tumour, which, in this series was removed and replaced by a titanium mesh. This was done both for oncological reasons and in anticipation of tumour necrosis following radiotherapy and consequent loss of contour. Large areas of mesh are likely to be unsightly, especially in exposed areas such as the forehead, but there is also a risk of erosion via skin, especially if radiation treatment was previously given or is anticipated. It is therefore useful to perform both MRI and CT to determine the likely extent and location of bone loss and consider alternative methods of bone replacement if the anticipated bone loss is extensive and/or in cosmetically critical areas. Several cranioplasty options exist and the selection of the optimum method should consider several factors. In meningioma, titanium is problematic as follow up imaging to detect recurrence is more difficult due to artefact [4, 20]. Furthermore, metalware can lead to protuberance, especially if radiation is also delivered through the overlying skin, leading to skin breakdown and/or discomfort for the patient [17]. Other materials such as hydroxyapatite can have problems with mechanical strength and may fracture easily [8].

The cost of cranioplasty is also a consideration [2]. This cost varies between different cranioplasty materials with metal-containing implants and those that require advanced design such as 3D printing proving more costly [2], although some manufacturing techniques have reduced these costs [7]. While EITC requires a hospital to have radiation therapy equipment, the only specific device required is a clinical linear accelerator and the cost associated with a single bone flap treatment is minimal. Despite the low cost for hospitals with radiation therapy capabilities, it is important to note that many low and middle-income countries do not have access to radiation therapy and the gap between demand and supply of this technology has widened over the past decade [32].

An ideal reconstructive technique would achieve the desired cosmetic effect, be biologically inert yet would readily integrate with the scalp and surrounding bone, mechanically strong and free of radiological artefacts, be delivered at the time of respective surgery, and cheap. While not fulfilling all these criteria for an ideal solution, based on this series of 18 cases as well as the other 23 published cases, EITC seems to be a safe and practical option.

### Limitations

All series of EITC for meningioma reported to date are retrospective. Multicentre prospective evaluation would be more rigorous at evaluating and comparing EITC outcomes with cranioplasties [2]. The duration of follow-up is another limitation of the series of EITC reported to date, including our own, which may underestimate specific long-term complications including tumour recurrence and bone resorption [28]. Furthermore, we observed that cosmetic outcome is not routinely documented at our institution, making it difficult to present the cosmetic outcomes of EITC in our cohort of patients. Metrics for functional and cosmetic outcomes following cranioplasty could be adapted for use in other reconstructive techniques such as EITC [11]. To determine whether irradiation of the flap after macroscopic removal of the tumour from the bone confers any additional benefit and to quantify it, an appropriate control group would be required.

## Conclusions

EITC for bone-invasive meningioma is a feasible and safe technique for managing meningioma-invaded bone. In the series presented here, we observed good outcomes and similar overall rates of recurrence and complications, including infection requiring explant compared to other reconstructive methods used in meningioma invading bone. Cosmetic outcome is universally under-reported and should be included in future studies alongside recurrence rates, complications, and other standard clinical outcomes. A prospective clinical trial comparing EITC and cranioplasty would be difficult to conduct given the small cohort of patients that may benefit from this approach. Systematic prospective collection of outcomes across multiple units, for example via a national or international registry, would provide further robust data on this technique.

## Data Availability

The corresponding author is happy to consider requests to use the study raw data.
